# Supplementary material for: The genome sequence of the Silver Birch, Betula pendula Roth (Fagales: Betulaceae)
Source: Wellcome Open Res. 2026 Sep 28;11:762. [Version 1] doi: 10.12688/wellcomeopenres.27951.1 (PMC13684027; doi:10.12688/wellcomeopenres.27951.1)
Supplement: Peer review history

# Reviewer report: Reviewer response for version 1

**DOI:** 10.21956/wellcomeopenres.30751.r172463

**Published:** 2026-10-21

**Authors:** Peng-Fei Ma

**Affiliations:** 1 Chinese Academy of Sciences, Kunming, China

In this study, the genome of silver birch, *Betula pendula*, was sequenced and assembled. This species is a deciduous tree and popular in gardens. The assembly is haplotype-resolved and comprises of 403 Mb and 411 Mb sequences at the chromosomal and scaffold level, respectively. Assembly quality assessment indicated a high quality for the assembled genome. In addition, the organelle genomes were also assembled and presented in the study.

Are the datasets clearly presented in a usable and accessible format, and the assembly and annotation available in an appropriate subject-specific repository?

Yes

Are sufficient details of the sequencing and extraction, software used, and materials provided to allow replication by others?

Yes

Are the rationale for sequencing the genome and the species significance clearly described?

Yes

Are the protocols appropriate and is the work technically sound?

Yes

Reviewer Expertise:

Plant genomics and evolution

I confirm that I have read this submission and believe that I have an appropriate level of expertise to confirm that it is of an acceptable scientific standard.

# Reviewer report: Reviewer response for version 1

**DOI:** 10.21956/wellcomeopenres.30751.r172462

**Published:** 2026-10-20

**Authors:** Himanshu Saini

**Affiliations:** 1 Adama Science and Technology University, Adama, Ethiopia

**Reviewer Report**

 The genome assembly of *Betula pendula (*Silver Birch) represents a significant advancement in plant genomics and genetic research. The successful assembly of two haplotypes, with genome sizes of 403.47 Mb and 411.74 Mb, demonstrates the high quality and comprehensiveness of the genomic data. The anchoring of 99.95% of haplotype 1 into 14 chromosomal pseudomolecules is particularly noteworthy. The assembly of mitochondrial and plastid genomes further enhances its scientific value. The identification of 29,794 protein-coding genes provides an important resource for functional genomics and evolutionary studies. Overall, this work makes a valuable contribution to the Darwin Tree of Life project and provides a robust foundation for future research on silver birch biology, biodiversity, and conservation.

Are the datasets clearly presented in a usable and accessible format, and the assembly and annotation available in an appropriate subject-specific repository?

Yes

Are sufficient details of the sequencing and extraction, software used, and materials provided to allow replication by others?

Yes

Are the rationale for sequencing the genome and the species significance clearly described?

Yes

Are the protocols appropriate and is the work technically sound?

Yes

Reviewer Expertise:

Plant Molecular Biology

I confirm that I have read this submission and believe that I have an appropriate level of expertise to confirm that it is of an acceptable scientific standard.

# Reviewer report: Reviewer response for version 1

**DOI:** 10.21956/wellcomeopenres.30751.r172468

**Published:** 2026-10-19

**Authors:** Nils Stein

**Affiliations:** 1 Leibniz Institute of Plant Genetics and Crop Plant Research (IPK), Seeland, Germany

Christenhuiz et al. report on the sequencing and assembly of the Betula pendula genome in frame of the DToL project, following established procedures, pipelines and data standards.

 Figure 1 is taken from Wikimedia, which is properly referenced, however, I have doubts that the species on the picture can be easily verified. For publications like this, providing reference datasets for the DToL project I personally consider it important if not essential to provide proper documentation of the sequenced sample. So if possible I recommend to change the picture.

 Figure 3 shows the HiC interaction plot on the chromosome scale assembly of haplotype one. Chromosome scaffolds 2, 6, 7, 8, 9 show suspicious interaction patterns that point to remaining assembly artifacts that should be subject to curation.

 while the authors report random display of chromosome order and orientation the Hic interaction plots would principally allow to suggest a proper order following general conventions (numbering of chromosomes from tallest to smallest and orienting from short to long arms. Due to the Rabl configuration most chromosomes can be ordered and oriented, in case there is no curated Betula karyotype available. I wonder whether DToL could not include a procedure into there standardized pipelines.

Are the datasets clearly presented in a usable and accessible format, and the assembly and annotation available in an appropriate subject-specific repository?

Yes

Are sufficient details of the sequencing and extraction, software used, and materials provided to allow replication by others?

Yes

Are the rationale for sequencing the genome and the species significance clearly described?

Yes

Are the protocols appropriate and is the work technically sound?

Yes

Reviewer Expertise:

plant biology, genetics, genomics

I confirm that I have read this submission and believe that I have an appropriate level of expertise to confirm that it is of an acceptable scientific standard, however I have significant reservations, as outlined above.

# Reviewer report: Reviewer response for version 1

**Published:** 2026-09-28

**Authors:** Automated Benchmarking

**Affiliations:** 1 Wellcome Sanger Institute, Hinxton, UK

As this article is a Genome Note, Automated Benchmarking has been used to determine whether the reported assembly passes four defined checks established by the Earth BioGenome Project: the raw and assembled data are publicly available, the Earth BioGenome Project three-part metric is met, the k-mer completeness exceeds 90%, and the BUSCO score passes the stated threshold. For Betula pendula (dhBetPend1), all four checks passed. Data available: pass. ENA study PRJEB89326, raw-read accessions ERR14955682 and ERR14986757, and assembly accessions GCA_965285875.1 and GCA_965285905.1 are public. Earth BioGenome Project three-part metric: pass. Haplotype 1 is reported as 6.C.Q64, meeting the 6.C.Q40 reference standard. k-mer completeness: pass. The combined haplotype assemblies have 98.97% k-mer completeness, exceeding the Earth BioGenome Project target of 90%. BUSCO: pass. Haplotype 1, which is proposed as the reference assembly for this species, has 98.5% complete BUSCOs (96.1% single-copy and 2.3% duplicated). BUSCO software version: 5.7.1; lineage dataset: eudicots_odb10. It meets the plant-specific target of greater than 90% complete BUSCOs. This decision uses values reported in the Genome Note and public accession records.

Reviewer Expertise:

NA

I confirm that I have read this submission and believe that I have an appropriate level of expertise to confirm that it is of an acceptable scientific standard.
